# Brown fat-specific mitoribosomal function is crucial for preventing cold exposure-induced bone loss

**DOI:** 10.1007/s00018-024-05347-4

**Published:** 2024-07-27

**Authors:** Jingwen Tian, Ji Sun Moon, Ha Thi Nga, Ho Yeop Lee, Thi Linh Nguyen, Hyo Ju Jang, Daiki Setoyama, Minho Shong, Ju Hee Lee, Hyon-Seung Yi

**Affiliations:** 1 Laboratory of Endocrinology and Immune System, Chungnam National University School of Medicine, Daejeon, 35015 Republic of Korea; 2 Department of Internal Medicine, Chungnam National University School of Medicine, Daejeon, 35015 Republic of Korea; 3 Department of Clinical Chemistry and Laboratory Medicine, Kyushu University Hospital, Fukuoka, Japan; 4 Graduate School of Medical Science and Engineering, Korea Advanced Institute of Science and Technology, Daejeon, Republic of Korea

**Keywords:** Cold exposure, Bone loss, Brown adipose tissue, Mitochondria

## Abstract

This study examines the interplay between ambient temperature, brown adipose tissue (BAT) function, and bone metabolism, emphasizing the effects of cold exposure and BAT mitochondrial activity on bone health. Utilizing ovariectomized (OVX) mice to model primary osteoporosis and BAT-specific mitochondrial dysfunction (BKO) mice, we evaluated the impact of housing temperature on bone density, immune modulation in bone marrow, and the protective role of BAT against bone loss. Cold exposure was found to universally reduce bone mass, enhance osteoclastogenesis, and alter bone marrow T-cell populations, implicating the immune system in bone remodeling under cold stress. The thermogenic function of BAT, driven by mitochondrial oxidative phosphorylation, was crucial in protecting against bone loss. Impaired BAT function, through surgical removal or mitochondrial dysfunction, exacerbated bone loss in cold environments, highlighting BAT’s metabolic role in maintaining bone health. Furthermore, cold-induced changes in BAT function led to systemic metabolic shifts, including elevated long-chain fatty acids, which influenced osteoclast differentiation and activity. These findings suggest a systemic mechanism connecting environmental temperature and BAT metabolism with bone physiology, providing new insights into the metabolic and environmental determinants of bone health. Future research could lead to novel bone disease therapies targeting these pathways.

**Supplementary Information:**

The online version contains supplementary material available at 10.1007/s00018-024-05347-4.

## Introduction

External temperature, as an environmental factor, influences numerous physiological processes, necessitating continuous adaptation of organisms to variations in temperature. Temperature variations can impact the functioning of the nervous system, [1] endocrine system, [2] and musculoskeletal system [3, 4]. Exposure to warm conditions have been shown to promote bone growth and enhance bone structure [3, 5, 6] Recently, the link between ambient temperature and bone density has garnered significant scientific attention. However, the full impact of environmental temperature on bone balance remains unclear. Certain studies have suggested that low temperature may increase bone density [4]. Conversely, other research has indicated that low temperature may adversely affect bone volume (BV), exemplifying the contrasting impacts of low temperature on bone health [3, 7]. Exposure to cold temperatures is also known to influence the production and release of neurotransmitters involved in the regulation of skeletal metabolism and endocrine functions [8, 9]. Additionally, a recent paper reported that thermoneutral housing of mice mitigates bone loss induced by OVX through the regulation of the gut microbiome environment [10]. Osteoporosis, a prevalent metabolic bone condition, is characterized by reduced bone density and the weakening of bone microarchitecture, [11] resulting in more fragile bones and an increased likelihood of fractures. The most common type of primary osteoporosis occurs as a result of post-menopausal estrogen deficiency, [12] and as such, it is exceedingly common in older women but can also occur in men. The impact of living temperature conditions during post-development and late adulthood, both in healthy individuals and those with osteoporotic conditions, on bone health, remodeling, and physiological processes remains uncertain.

Maintaining a constant body temperature in a changing temperature environment is critical for survival. When exposed to low temperatures, mammals increase oxidative metabolism for heat production. BAT is the main site of non-shivering thermogenesis in mammals. BAT volume has been reported to correlate with bone mass, [13] to influence positively bone mass, and to serve as a reliable indicator of the structural integrity of the femur, [14] studies in human and mice have identified brown adipose tissue as a significant independent factor influencing bone mass [4, 15]. Additionally, A previous study reported that BAT has been shown to rescue bone loss induced by cold exposure at an early time point, [16] BAT-deficient mice show a marked reduction in bone formation [17]. However, Although previous studies have shown a correlation between BAT and bone mass, the exact mechanism by which BAT affects bone mass is not fully understood.

CR6-interacing factor 1 (CRIF1) is a protein that plays a crucial role in mitochondrial function and essential for the synthesis and integration of mitochondrial oxidative phosphorylation (OxPhos) subunits within the mitochondrial inner membrane [18]. Mice with a tissue-specific deficiency of *Crif1* have markedly impaired mitoribosome-mediated translation [18–21]. BAT mitochondrial oxidative activity is increased by cold exposure [22]. Brown adipocytes are characterized by their abundant mitochondria, which possess a high capacity for oxidation, and contain UCP-1 within their inner membranes [23]. Thus, we hypothesized that mitochondrial dysfunction in BAT would prevent its activation in response to cold exposure. Therefore, we considered the BAT-specific mitochondrial dysfunction mouse model to be suitable for investigating the mechanisms underlying bone loss induced by cold exposure and the role of BAT in this process.

## Materials and methods

### Mice

*Ucp1-cre* transgenic (Tg [*Ucp-Cre*]1Evdr) mice were purchased from Jackson Laboratory, and were backcrossed to C57BL/6J background mice. Floxed *Crif1* (*Crif1*^*f/f*^) mice were generated as previously described [18]. The mice in this study were kept in a specific pathogen-free environment at the Preclinical Research Center of Chungnam National University Hospital and were housed under standard conditions, which included a cycle of 12 h of light followed by 12 h of darkness, a stable room temperature of 22 °C, and a relative humidity of between 40 and 60%. Their diet consisted of Teklad global chow with 18% protein content (2918 C, ENVIGO). Based on studies of mouse metabolic rates at different temperatures, temperatures of 28–33 °C are regarded as thermoneutral housing conditions for mice, while 28–16 °C is considered mild cold, and 16–5 °C is considered severe cold [24]. Below thermoneutral conditions, mice require additional energy for thermogenesis to maintain their body temperature [25]. To investigate the effects under various temperature conditions, the mice were maintained at 22 °C, which corresponds to standard room temperature housing conditions and is designated as a mild cold condition, at 30 °C, which corresponds to thermoneutral housing conditions, and at 14 °C, designated as a severe cold condition. in a light and humidity-controlled chamber. All experimental procedures complied with the guidelines of Institutional Animal Care and Use Committees (IACUCs).

### OVX mice and the BAT-deficient mouse model

Female C57BL/6J mice (8 weeks old) were anesthetized and a 50 mm incision was made on their backs. Ovaries on both sides were exposed and removed after tubal ligation, and the wound was sutured. Sham group animals underwent a similar procedure but the ovaries were not removed. Male C57BL/6J mice (8 weeks old) were anesthetized, their skin incised, and the interscapular BAT was completely excised.

### Micro-CT analysis

Micro-CT was performed on vertebrae and long bones using SkyScan 1173 (SkyScan, Belgium) with 8 μm resolution. All bone morphometric parameters were calculated three-dimensionally with CTan and CTvox version 1.6, which was used to measure BV, total volume, BV/TV, bone surface, bone surface density, trabecular thickness, and trabecular separation. All bone micro-CT nomenclature followed the guidelines of the American Society for Bone and Mineral Research (ASBMR).

### RNA extraction and real-time PCR analysis

RNA was isolated from BAT using the TRIzol method (Life Technologies, Eugene, OR, USA). The extracted RNA was then converted into complementary DNA (cDNA) using M-MLV reverse transcriptase and oligo-dT primers (Invitrogen, Carlsbad, CA, USA). For amplification of specific gene sequences, each cDNA sample was processed using SYBR Green PCR Master Mix (Applied Biosystems, Foster City, CA, USA) and appropriate primers in a 7500 Fast Real-Time PCR System with Software version 2.0.6 (Applied Biosystems). The relative gene expression levels were quantified using the ΔΔCT method with Applied Biosystems 7500 Software (version 2.0.6), and normalization was done against the 18 S housekeeping gene. Primer sequences are shown in Table 1.

### Western blot analysis

Tissue samples were homogenized by Tissue lyser II (Qiagen, Venlo, Netherlands) using protein lysis buffer (50 mM Tris-HCl, pH 7.4, 1 mM EDTA, 150 mM NaCl, 0.1% Triton X-100) with protease inhibitor cocktail (#11,836,145,001, Roche, Basel, Switzerland) and phosphate inhibitor (#04906837001, Roche). After homogenization, samples were centrifuged at 13,000 rpm for 15 min, and the supernatant was collected. To obtain pure protein samples, samples were centrifuged again. Protein concentrations were measured using the BCA protein assay (#23,227, Thermo Fisher Scientific). Proteins (20 µg) were loaded on a sodium dodecyl sulfate-polyacrylamide gel and separated according to standard methods. The proteins were transferred to a 0.45 µM pore nitrocellulose membrane (Amersham Bioscience, Germany). Blocking was performed using 5% skim milk with TBST buffer for 1 h. Membranes were incubated with primary antibody overnight. After washing, the membranes were incubated with secondary antibodies for 2–4 h. Secondary antibodies were purchased from Santa Cruz Biotechnology and Cell Signaling Technology. ECL solution (Advansta, USA) was spread to the membranes to detect immunoreactive protein bands, and images were obtained using the ODYSSEY instrument and Image StudioTM software (Li-COR Biosciences, Lincoln, NE, USA). The antibodies used are listed in Table 2.

### Serum measurement

Blood samples were collected by cardiac puncture of mice under general anesthesia. Samples were centrifuged at 10,000 rpm for 5 min. Serum levels of RANKL and free fatty acid were determined using specific enzyme-linked immunosorbent assays following the manufacturer’s protocols.

### In vitro osteoclastogenesis

Bone marrow cells were harvested from the femurs of 6–8-week-old C57BL/6J mice using a sterile 21-gauge needle for flushing. These cells were then cultured in alpha-MEM supplemented with 10% FBS and 30 ng/mL of macrophage colony-stimulating factor (M-CSF) (R&D Systems, Minneapolis, MN, USA). After 2 days, the adherent cells, corresponding to differentiated bone marrow-derived monocyte/macrophages, were further incubated with 50 ng/mL of RANKL and 10 ng/mL of M-CSF, with or without the addition of palmitic acid, to facilitate osteoclast formation. Following a 3-day culture period, the cells were fixed and subjected to TRAP staining using a kit (Sigma-Aldrich, St. Louis, MO, USA).

### T-cell activation

Bone marrow cells were isolated from femurs of 8-week-old C57BL/6J mice. The cells were seeded in 24-well plates and cultured in RPMI-1640 medium containing 10% FBS. After 24 h, floating cells were collected and stimulated with Cell Stimulation Cocktail (eBioscience, San Diego, CA, USA) for 5 h and harvested. The cells were fixed and permeabilized using Fixation/Permeabilization Buffer kit (eBioscience, San Diego, CA, USA), and stained for intracellular cytokines with anti-CD254 PE and anti- IFN-γ APC antibodies. Multicolor flow cytometry analysis was conducted using the LSRFortessa flow cytometer (BD Biosciences, NJ, USA), and the resulting data were processed and interpreted with the aid of FlowJo software (Tree Star, Ashland, OR, USA).

### Flow cytometry analysis

Isolated bone marrow cells were passed through a 70-µm cell strainer, rinsed with phosphate-buffered saline, and then resuspended in 40% Percoll solution (GE Healthcare, Chalfont St Giles, UK). The suspension was subjected to centrifugation at 2,400 rpm for 30 min at a temperature of 4 °C. Subsequently, the cells were treated with fluorochrome-labeled monoclonal antibodies for a duration of 60 min at 4 °C. The antibodies used in this study are listed in Table 2. To prevent non-specific antibody attachment, cells were initially treated with an anti-mouse CD16/32, which blocks mouse Fc receptors. (BD Biosciences, San Jose, CA, USA) Before applying specific antibodies for staining, bone marrow cells were pre-treated with a Cell Stimulation Cocktail (eBioscience, San Diego, CA, USA) to facilitate intracellular staining for 5 h. The cells were fixed and permeabilized using a Fixation/Permeabilization Buffer kit (eBioscience, San Diego, CA, USA), and then washed and resuspended in 1% formaldehyde, and further stained for intracellular cytokines with anti-IFN-γ-APC, anti-CD254-PE, or anti-IL-17 A-APC antibody. Foxp3 staining was performed after fixation and permeabilization using the eBioscience Foxp3 staining kit.

### Bone histological analysis

For the examination of bone histology, the femurs and tibias of the mice were collected, cleared of skin, and preserved in 4% paraformaldehyde overnight at ambient temperature. Subsequently, the samples were dehydrated using an ethanol solution and were decalcified with 10% solution of ethylenediaminetetraacetic acid (Sigma-Aldrich, Dorset, UK) for 4 weeks at room temperature. The buffer was changed every 3–4 days until complete decalcification. The tissues were embedded in paraffin, and 4-µm sagittal-oriented sections were prepared and stained with H&E and TRAP for histological analysis using standard protocols. For von Kossa staining, undecalcified bones were embedded in methyl-methacrylate (Sigma) and 6-µm-thick sections were stained with von Kossa reagent.

### Histological analysis

BAT and iWAT tissues were fixed in 4% paraformaldehyde (PFA) for 24 h. Following dehydration in ethanol, the samples were embedded in paraffin. For hematoxylin and eosin staining, sections with a thickness of 4 μm were deparaffinized with xylene and rehydrated through a graded series of ethanol to water. The sections were then stained with hematoxylin for 4 min followed by eosin staining for 2 min. Immunohistochemistry was performed according to standard protocols.

### LC-MS measurements

Plasma samples were analyzed using LC-MS on an LCMS-8060 instrument. To analyze a broad spectrum of water-soluble metabolites, the prepared samples underwent separation on a Discovery HS-F5-3 column with specific dimensions and particle size using two solvent phases with distinct compositions. The column was maintained at a controlled temperature. The column was eluted using a gradient elution program, with flow rates and solvent compositions varying over time. The heated electrospray ionization (ESI) source operated in both negative and positive ion modes, with multiple reaction monitoring, and parameters such as gas flow rates, temperatures, and pressures were carefully set for optimal results.

For the separation of acylcarnitines, a different column was used with predefined mobile phases and temperature settings. The gradient elution program was adjusted to cater for the specific requirements of acylcarnitine separation. Detection was carried out in positive ESI mode, with the collision energy adjusted according to the lengths of the fatty acids.

For the separation of free fatty acids, separation was achieved using an ACQUITY BEH Amide column, with a specific mobile phase composition and temperature. The gradient elution program was tailored for free fatty acids, and detection was conducted in negative ESI mode with selected ion monitoring.

Bile acids were separated using an ACQUITY BEH Amide column, but with different solvent compositions and a higher column temperature. The gradient elution program was specifically designed for bile acids, and detection was done in negative ESI mode with multiple reaction monitoring.

Phospholipids were separated on a Kinetex C8 column, with a unique combination of solvents and a specific temperature setting. The gradient elution program was carefully planned, and phospholipid profiles were detected in positive ESI mode with multiple reaction monitoring.

### Statistical analysis

All Statistical analyses were conducted using GraphPad Prism software (version 9, Dotmatics, San Diego, USA). Data were expressed as the mean ± SD. Unpaired Student’s t-tests and one-way ANOVA followed by Scheffe’s post-hoc test were used to determine statistical significance, with a *p*-value of less than 0.05 considered significant.

## Results

### Low temperature accelerates bone loss in mice

**Fig. 1:**
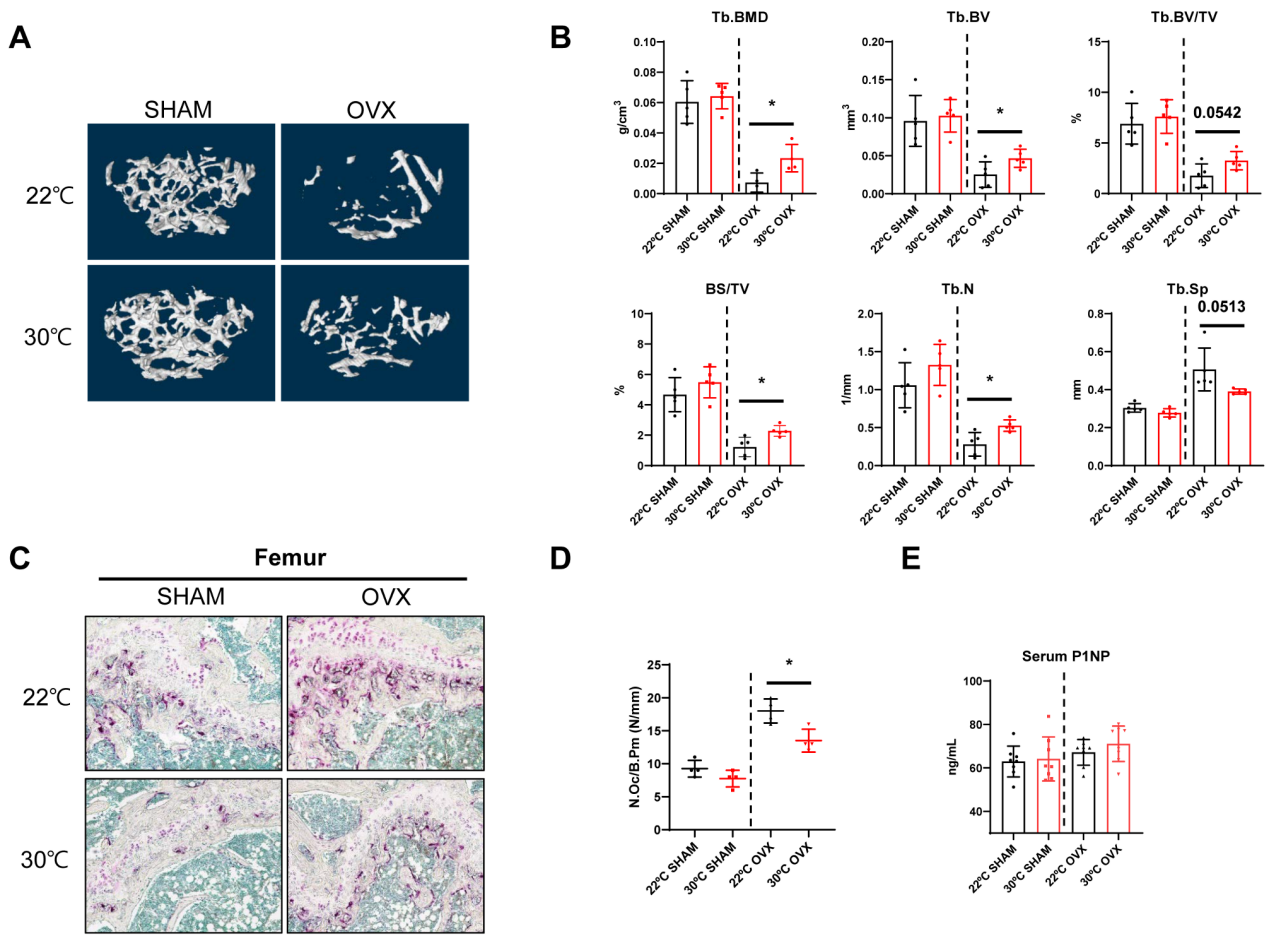
Aggravation of ovariectomy-induced bone loss by low temperature housing conditions. **A** Illustrative micro-CT images showing the trabecular areas in the lower section of the femur. **B** Micro-CT measurement of trabecular bone mineral density (Tb.BMD); trabecular bone volume (Tb.BV); trabecular bone volume/tissue volume (Tb.BV/TV); trabecular bone surface/tissue volume (Tb.BS/TV); trabecular number Tb.N; and trabecular separation Tb.Sp **C**,** D** TRAP staining for osteoclasts and statistical analysis of osteoclast number. E, Serum levels of P1NP. The results were expressed as the mean ± SD. Statistical significance was estimated using unpaired t-tests. *, *P* < 0.05

To explore the effects of housing temperature on bone mass homeostasis, we employed micro-computed tomography (micro-CT) analysis to evaluate the bone structural features of SHAM and OVX mice, a commonly used primary osteoporosis model. After performing OVX and SHAM surgery on 8-week-old mice, the mice were maintained at 22 °C, corresponding to standard room temperature housing conditions, which we designated as a mild cold condition, and 30 °C, corresponding to thermoneutral housing conditions, for 12 weeks, and the experiments were performed when the mice were 20 weeks old. Micro-CT analysis was conducted to assess cortical and trabecular bone architecture of SHAM and OVX mice housed under room temperature conditions and those housed under thermoneutral conditions. As expected, in trabecular bone, thermoneutral housing conditions significantly increased bone mineral density (BMD), BV, and trabecular number in OVX mice but not in SHAM mice (Fig. 1A and B). OVX not only induces osteoporosis due to estrogen deficiency but is also known to impair estrogen-mediated BAT activation [26, 27]. We hypothesize that the acceleration of bone loss due to cold exposure observed only in OVX mice, and not in SHAM mice, may be attributed to the reduction in the protective effects of estrogen-mediated BAT activation. In cortical bone, housing temperature had no effect on these parameters in either SHAM or OVX mice (Supplementary Fig. 1A and 1B). Next, we examined the bone phenotypes under severe cold housing conditions at temperatures below 16 °C (14 °C). The SHAM mice housed under these conditions showed decreases in trabecular and cortical bone parameters compared with those housed under room temperature conditions. In OVX mice housed under severe cold conditions, trabecular BMD was significantly lower than in those housed under room temperature conditions (Supplementary Fig. 2A and 2B). Tartrate-resistant acid phosphatase (TRAP) staining revealed that the number of mature osteoclasts was significantly higher in OVX mice than in SHAM mice and significantly higher in OVX mice housed at 22 °C than in those housed at 30 °C (Fig. 1C and D). However, there was no significant difference in the serum level of P1NP, a bone formation marker, between mice housed at 22 °C and those housed at 30 °C (Fig. 1E). Collectively, exposure to lower temperature appears to induce bone loss accompanied by an increase in the number of osteoclasts.

### Temperature-dependent modulation of bone marrow T-cells and BAT function

**Fig. 2:**
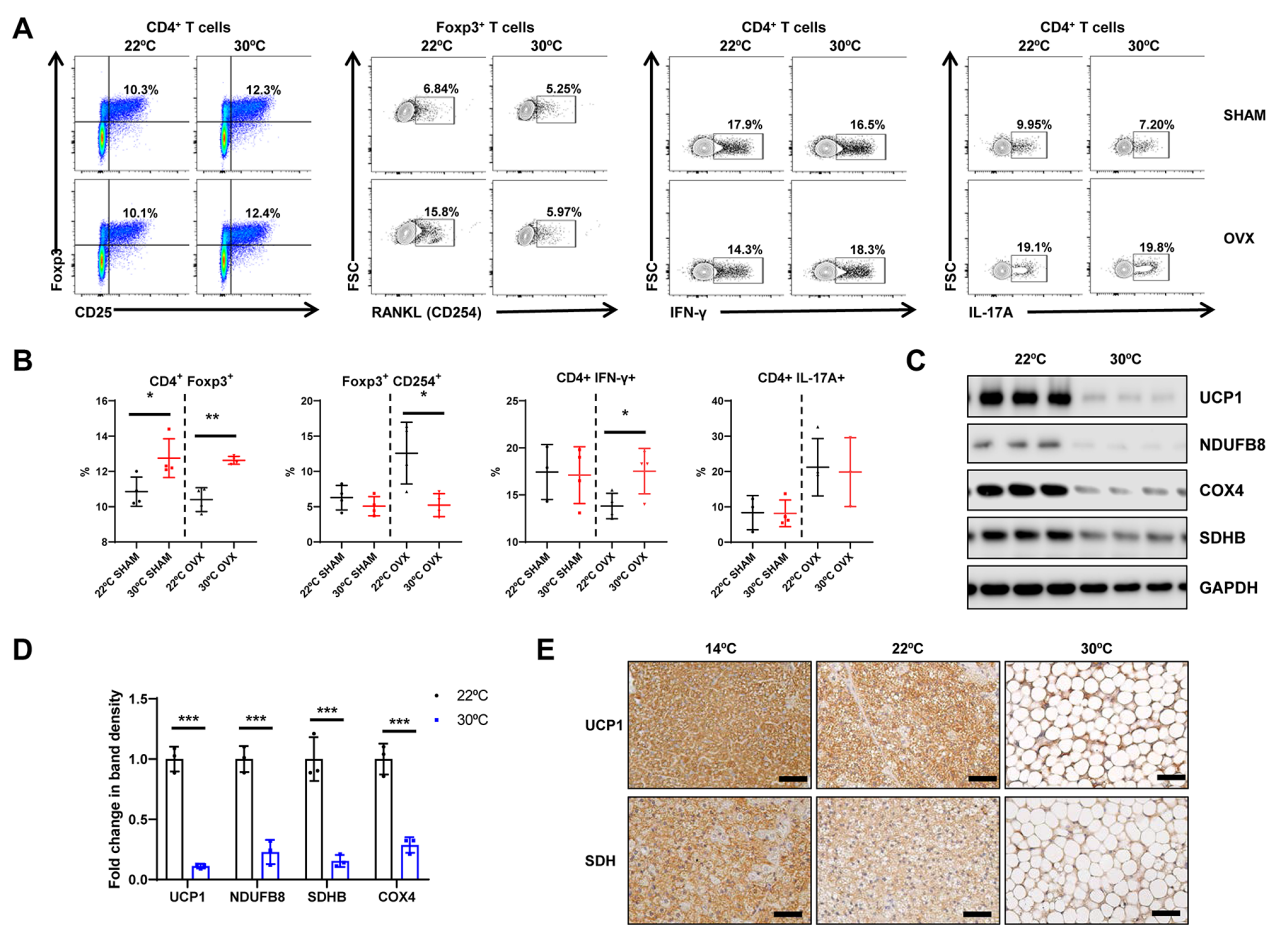
Temperature-mediated changes in osteoclastogenic bone marrow T cells and brown adipose tissue (BAT). **A** Representative contour plots from flow cytometry for regulatory T-cells (Treg; CD4 + CD25 + FOXP3+); RANKL (CD254) producing Treg cells; and IFN-γ or IL-17 A-producing CD4 + cells from bone marrow. **B** Statistical analysis of phenotypes defined by flow cytometry. **C**,** D** Representative western blots and band density measurements of UCP-1 and OxPhos complex subunits in the BAT isolated from mice housed for 12 weeks at 22–30 °C. **E** Representative immunohistochemical images of UCP-1 and SDH staining of BAT from mice housed at different temperatures. Scale bar, 50 μm. Data were expressed as the mean ± SD. Statistical significance was estimated using unpaired t-tests. *, *P* < 0.05, **, *P* < 0.01 ***, *P* < 0.001

Next, we investigated how housing temperature affects bone marrow immune cell phenotypes, which is an important factor in the initiation of bone resorption [21]. To address this issue, we examined the expression of osteoclastogenic cytokines in bone marrow cells from mice housed at 22 °C and 30 °C. We observed a decrease in the populations of CD4 + CD25 + Foxp3 + regulatory T-cells (Tregs) in both OVX and SHAM mice at 22 °C. Previous studies showed that Tregs produce RANKL, which is important for osteoclastogenesis and is linked to the bone damage observed in inflammatory arthritis [28]. We found that the expression of RANKL (CD254^+^) in Tregs was higher in OVX mice housed at 22 °C than in those housed at 30 °C. The expression of IFN-γ, a cytokine known to inhibit osteoclast differentiation, was lower in the CD4 + cells of OVX mice housed at 22 °C than in those housed at 30 °C. The expression of IL-17 A + was higher in CD4 + cells of OVX mice than in those of SHAM mice, irrespective of housing temperature (Fig. 2A and B). These changes subsequently regulate the proliferation of osteoclasts. Additionally, we examined changes in BAT, an organ known for its temperature sensitivity. As reported previously, at lower temperatures, BAT exhibited significantly higher expression of UCP-1 and mitochondrial OxPhos complex subunits, including complex I (NDUFB8), II (SDHB), and IV (COX4) (Fig. 2C and D). Immunohistochemistry also revealed higher UCP-1 and succinate dehydrogenase (SDH) expression in BAT at lower temperatures in SHAM mice (Fig. 2E).

### Impact of BAT removal on bone loss

**Fig. 3:**
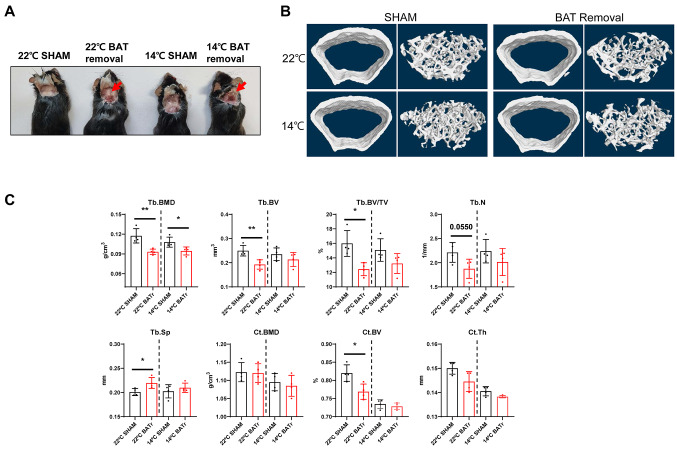
Removal of interscapular BAT promotes bone loss. **A** Images of mice at 12 weeks after post-surgical removal of BAT. **B** Representative images of micro-CT of cortical and trabecular regions in the distal femur. **C** Measurement of Tb.BMD, Tb.BV, Tb.BV/TV, Tb.N, Tb.Sp, Ct.BMD, Ct.BV, and Ct.Th in the femur of SHAM and BAT-deficient mice housed at 22 °C–14 °C for 12 weeks. Statistical significance was estimated using unpaired t-tests. *, *P* < 0.05, **, *P* < 0.01

Next, we investigated whether BAT is directly involved in bone loss caused by cold exposure. At 8 weeks of age, mice were randomly assigned to undergo either a sham operation (SHAM mice) or surgical removal of interscapular BAT (BAT-deficient mice). Subsequently, the SHAM and BAT mice were housed at temperatures of either 22 °C–14 °C for 12 weeks. The BAT-deficient mice housed at either of these temperatures did not show BAT regeneration at the end of the 12-week period. (Fig. 3A). The BAT mice housed at 22 °C showed decreases in trabecular BMD, trabecular BV, and trabecular percent BV, and an increase in trabecular separation. Cortical BV was also decreased in the cortical bone region. The BAT mice housed at 14 °C also tended to show decreases in bone parameters, but the decreases were not statistically significant (Fig. 3B and C), with the exception of trabecular BMD. These findings lend support to the hypothesis that surgical removal of BAT exacerbates bone loss induced by cold exposure.

### BAT-specific mitochondrial OxPhos dysfunctional mouse model

**Fig. 4:**
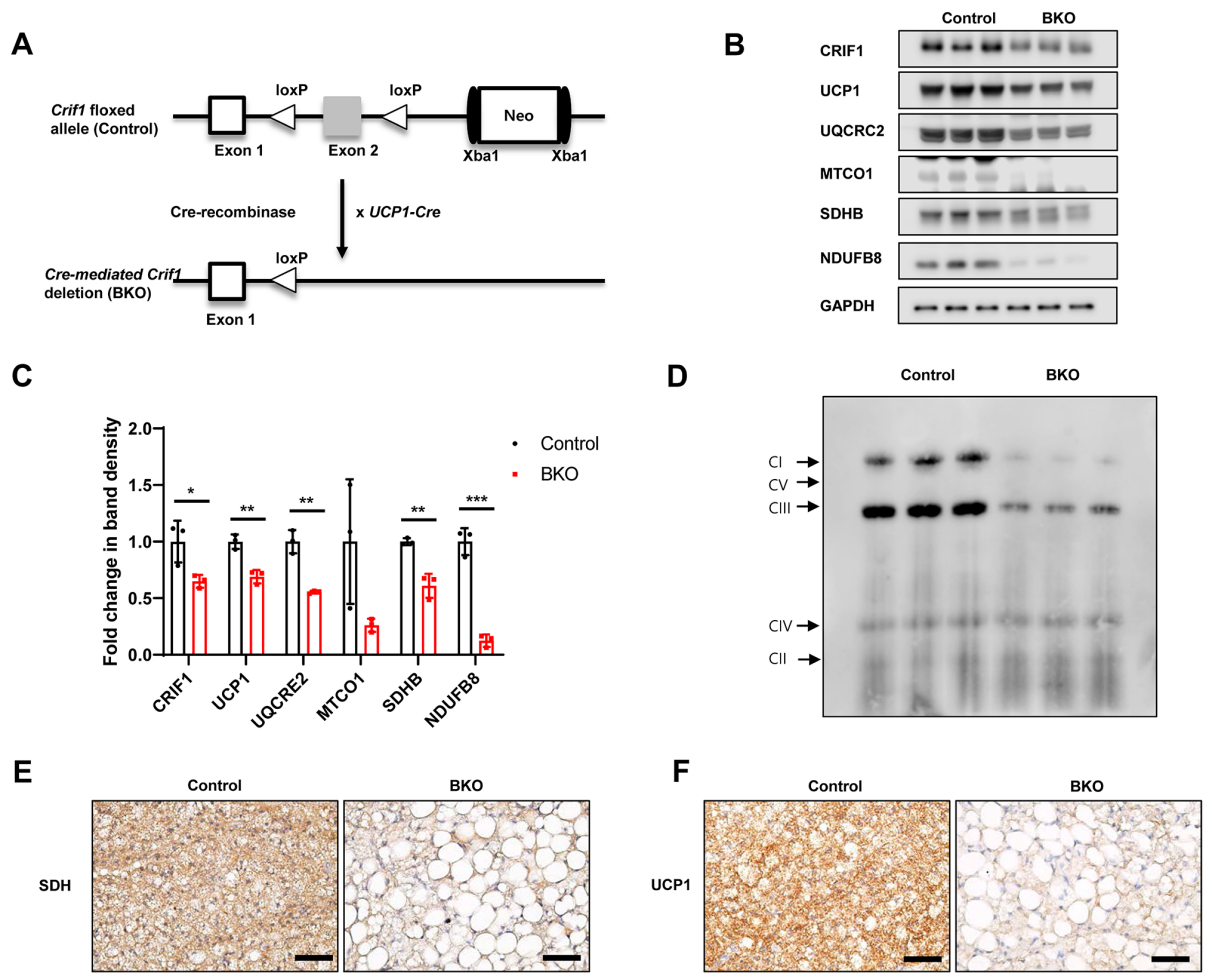
BAT-specific mitochondrial dysfunction (BKO) mice show impairment of OxPhos. **A** BKO mice generated by knockout of *Crif1* in brown adipose tissue using UCP-1-cre mice and the *Cre-loxP* system. **B**,** C** Immunoblotting of OxPhos complex subunits and UCP-1 in BAT isolated from control and BKO mice housed at 22 °C at 20 weeks old. **D** Representative BN-PAGE image of the assembled OxPhos complex in BAT tissue. **E**,** F** Representative immunohistochemical images of UCP-1 and SDH staining in BAT paraffin sections. Scale bar, 50 μm. The statistical relevance of the findings was determined using unpaired t-tests. *, *P* < 0.05, **, *P* < 0.01 ***, *P* < 0.001. The analysis was conducted in comparison with the specified reference group

To further determine the impact of BAT mitochondrial OxPhos dysfunction on the bone, we generated BAT-specific *Crif1* knockout (BKO) mice through selective disruption of *Crif1* in brown adipocytes using the Cre-loxP system. *Crif1*-floxed (*Crif1*^*f/f*^) mice were bred with *Ucp1-Cre* transgenic mice, resulting in the deletion of exon 2 of the *Crif1* gene (Fig. 4A). *Crif1* deficiency resulted in reduced translation of CRIF1, OxPhos subunits, including complex I (NDUFB8), complex II (SDHB), complex III (UQCRC2), and complex IV (MTCO1), and decreased UCP-1 expression in mice housed under chronic cold (22 °C) conditions (Fig. 4B and C). Blue native-PAGE (BN-PAGE) analysis of mitochondria isolated from BAT revealed reduced levels of native OxPhos complexes I and III (Fig. 4D). Immunohistochemistry staining showed that BAT from the *Crif1*-knockout had reduced succinate dehydrogenase (SDH) expression, confirming reduced mitochondrial oxidative phosphorylation in the OxPhos dysfunctional mice, and reduced UCP-1 expression (Fig. 4E and F). To exclude off-target effects in BKO mice, we examined the expression of CRIF1 in the liver, muscle, heart, and iWAT of BKO mice and control mice (Supplementary Fig. 3A and 3B). There was no significant difference in CRIF1 expression between the control and BKO mice, confirming the specificity of the knockout. Taken together, these findings imply that *Crif1* deficiency in BAT sufficiently impairs OxPhos dysfunction in mice.

### Mitochondrial oxidative phosphorylation dysfunction in BAT accelerates bone loss caused by chronic cold stress

**Fig. 5:**
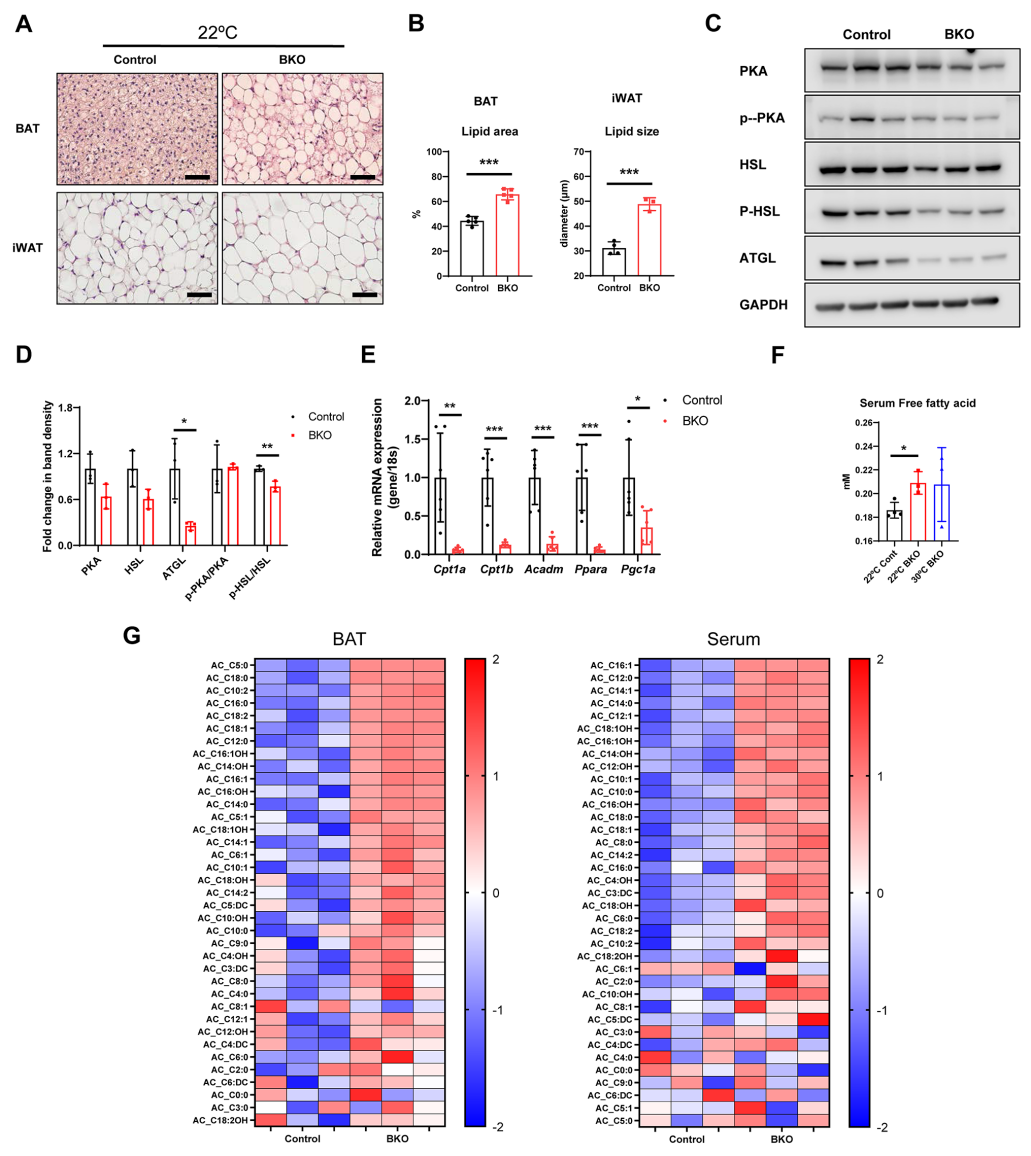
BAT-specific mitochondrial dysfunction (BKO) mice exposed to cold stress exhibit bone loss and an osteoclastogenic bone marrow T-cell phenotype. **A** Micro-CT scans showing the cortical and trabecular structures in the distal part of the femur. **B** Measurement of Tb.BMD, Tb.BV/TV, Tb.Th, Tb.N, Tb.Sp, Ct.BMD, Ct.BV and Ct.Th in the femur. **C** Representative contour plots from flow cytometry for regulatory T-cells (Treg; CD4 + CD25 + FOXP3+); RANKL (CD254) producing Treg cells; and IFN-γ producing CD4 + cells from bone marrow. **D** Serum levels of RANKL. Data are expressed as the mean ± SD. Statistical significance was determined using one-way ANOVA for comparative analysis. *, *P* < 0.05 and **, *P* < 0.01 ***, *P* < 0.001

Loss of mitochondrial function in BAT is expected to result in multiple phenotypes upon cold exposure. Thus, we hypothesized that cold-induced activation of BAT would be compromised in BAT with mitochondrial oxidative phosphorylation dysfunction, leading to bone loss. To validate this hypothesis, we assessed the bone parameters of BKO mice at 20 weeks of age using micro-CT. In the trabecular region, the BKO mice housed at 22 °C exhibited significant decreases in BMD, BV, percent BV, trabecular number and thickness, and an increase in trabecular separation (Fig. 5A and B) compared with control mice housed under the same condition. Additionally, cortical BMD, BV, and thickness (Fig. 5A and B) were lower in these mice than in the control mice. Furthermore, BKO mice maintained at 30 °C for 12 weeks started to show reversal of bone loss in the trabecular region at 8 weeks of age (Fig. 5A and B). Additionally, while there was no pronounced increase in BMD in cortical bone of BKO mice maintained at 30 °C for 12 weeks, there was a discernible improvement in cortical BV and thickness (Fig. 5A and B). At a housing temperature of 22 °C, BKO mice consistently displayed lower trabecular BMD and BV (Supplementary Fig. 4A and 4B) than control mice. Moreover, von Kossa staining showed decreased bone parameters not only in the femur but also in the vertebrae (Supplementary Fig. 4C). Taken together, these results indicate that BKO mice exhibit bone loss when exposed to chronic cold stress (22 °C), but under thermoneutral conditions (30 °C) have bone parameters similar to those of wild-type mice housed under the same conditions. This suggests that the activation of BAT mitochondria during cold exposure plays a pivotal role in maintaining bone mass.

Next, we investigated whether mitochondrial dysfunction in BAT affects the bone marrow immune environment, which is a critical determinant for the initiation of bone resorption, and further determined whether mitochondrial OxPhos dysfunction in BAT triggers osteoclastogenesis in bone marrow. To do this, we quantified various T-cell populations in the BM of control and BKO mice by flow cytometry analysis. We found that at 20 weeks of age, Treg populations were significantly larger in the bone marrow of BKO mice (Fig. 5C) than in that of control mice, Additionally, a subset of the Treg cell population expressing RANKL was also higher in BKO mice than in control mice (Fig. 5C). The serum level of RANKL was also increased in these mice (Fig. 5D). Furthermore, in the CD4 + T-cell population, IFN-γ expression, which is known to inhibit osteoclast differentiation, was lower in BKO mice than in control mice (Fig. 5C). Thus, at low temperatures (22 °C) requiring thermogenesis, BKO mice with BAT mitochondrial dysfunction have a bone marrow immune environment that favors osteoclastogenesis. Under thermoneutral conditions (30 °C), the expression of RANKL in Treg cells and IFN-γ in CD4 + cells was lower than that in the controls (Fig. 5C).

### Alterations in lipid composition within thermogenic adipose tissue and corresponding metabolite variations in serum

**Fig. 6:**
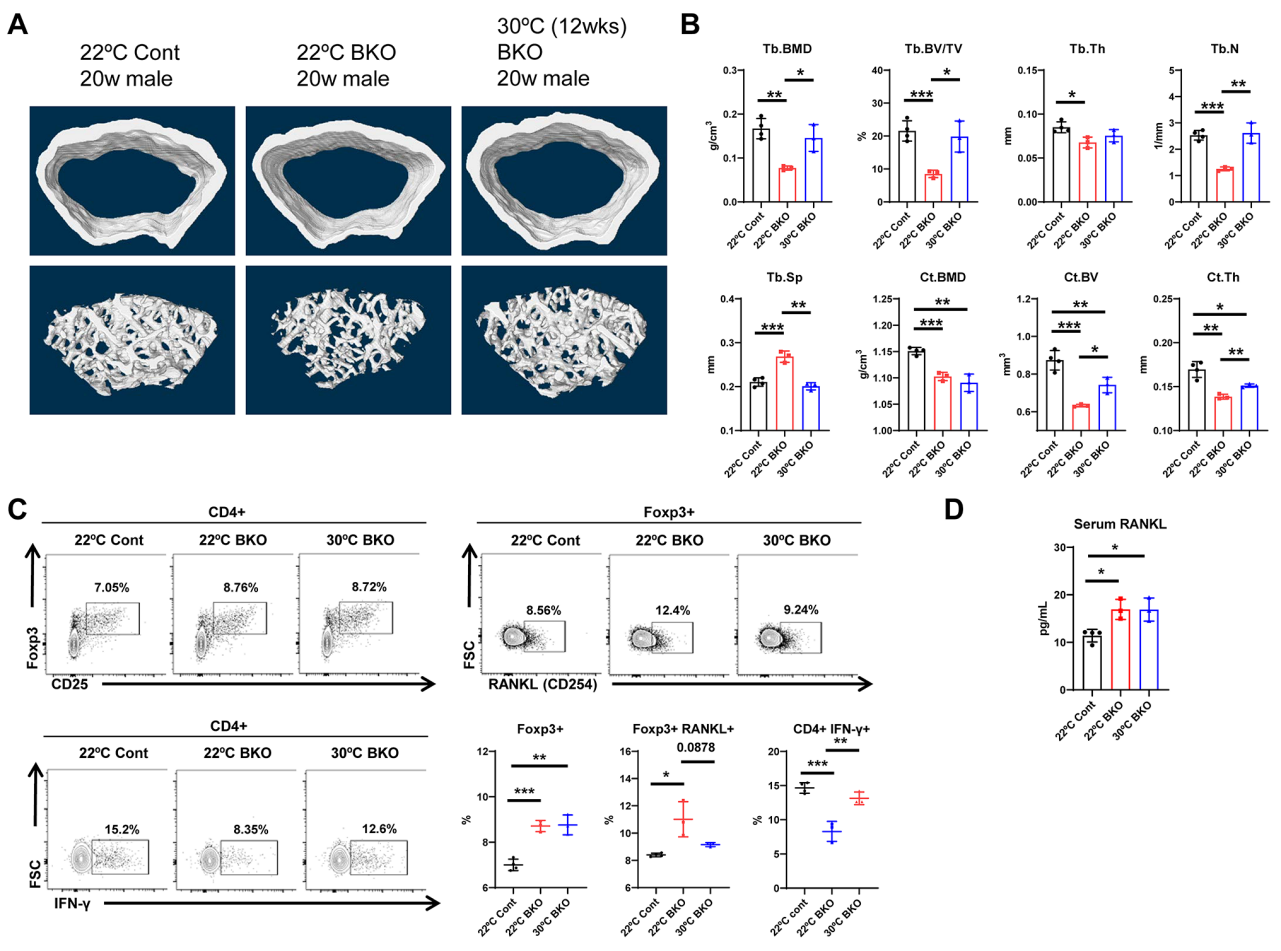
Modifications of lipid composition in thermogenic adipose tissue. **A** Representative images of H&E staining of BAT and iWAT sections obtained from 20-week-old mice housed at 22 °C. Scale bar, 50 μm. **B** Statistical analysis of lipid area in BAT and lipid diameter in iWAT. **C**,** D** Representative western blots and band density measurements for PKA, p-PKA, HSL, p-HSL and ATGL in BAT of the control and BAT-specific mitochondrial dysfunction (BKO) mice. **E** Real-time PCR analysis of fatty acid oxidation related genes in the BAT of 20-week old control and BKO mice. **F** Quantification of serum free fatty acids. **G** Comparative analysis of BAT and serum metabolite levels in 20-week-old control and BKO mice. Statistical significance was analyzed by one-way ANOVA. *, *P* < 0.05 and **, *P* < 0.01 ***, *P* < 0.001 compared with the indicated group

Brown fat tissue is a site of active lipid catabolism, resulting in the release fatty acids for energy utilization via lipolysis. These fatty acids fuel thermogenesis, a process in which energy is not converted into ATP but instead released as heat. This is critical for body temperature regulation, especially during cold stress. This thermogenic response is orchestrated by UCP-1, which is prevalent in the mitochondrial membrane of brown fat cells and disrupts the typical pathway of oxidative phosphorylation [29]. In this study, we observed a decrease in the lipid area within BAT when the mice were housed at low temperatures (22 °C and 14 °C) (Supplementary Fig. 5A and 5B). Furthermore, consistent with previous reports, [30] Western blot analysis confirmed that cold temperature housing conditions activate lipolysis (Supplementary Fig. 5C and 5D), Additionally, we confirmed that the surgical removal of BAT led to an increase in serum free fatty acid levels (Supplementary Fig. 5E), suggesting that BAT may counteract low temperatures by inducing thermogenesis using the energy derived from lipolysis. Next, we hypothesized that BAT in BKO mice would exhibit impaired lipolysis and fatty acid oxidation. To test this hypothesis, we examined the BAT phenotype of BKO mice. The BKO mice had larger lipid areas in BAT and larger lipid droplet sizes in inguinal white adipose tissue (iWAT) (Fig. 6A and B). Additionally, Western blot analysis revealed reduced lipolysis in BAT (Fig. 6C and D) and RT-PCR revealed reduced expression of genes controlling fatty acid oxidation (*Cpt1a*,* Cpt1b*,* Acadm*,* Ppara*, and *Pgc1a*) (Fig. 6E). Furthermore, BKO mice had elevated serum levels of free fatty acids (Fig. 6F), which may be attributed to decreased lipolysis and fatty acid oxidation in these mice. As shown in Fig. 6G, the increased metabolites in the BAT and serum of BKO mice were predominantly fatty acids, including both saturated and unsaturated fatty acids, with a notable increase in long-chain fatty acid levels in serum. Collectively, dysfunctional BAT mitochondria impair BAT fatty acid oxidation, leading to elevated long-chain fatty acids in the bloodstream. Furthermore, the increased levels of circulating free fatty acids are likely responsible for the observed increase in lipid size within the iWAT (Fig. 6A).

### Long-chain fatty acids activate osteoclasts and promote T-cell osteoclastogenic transformation

**Fig. 7:**
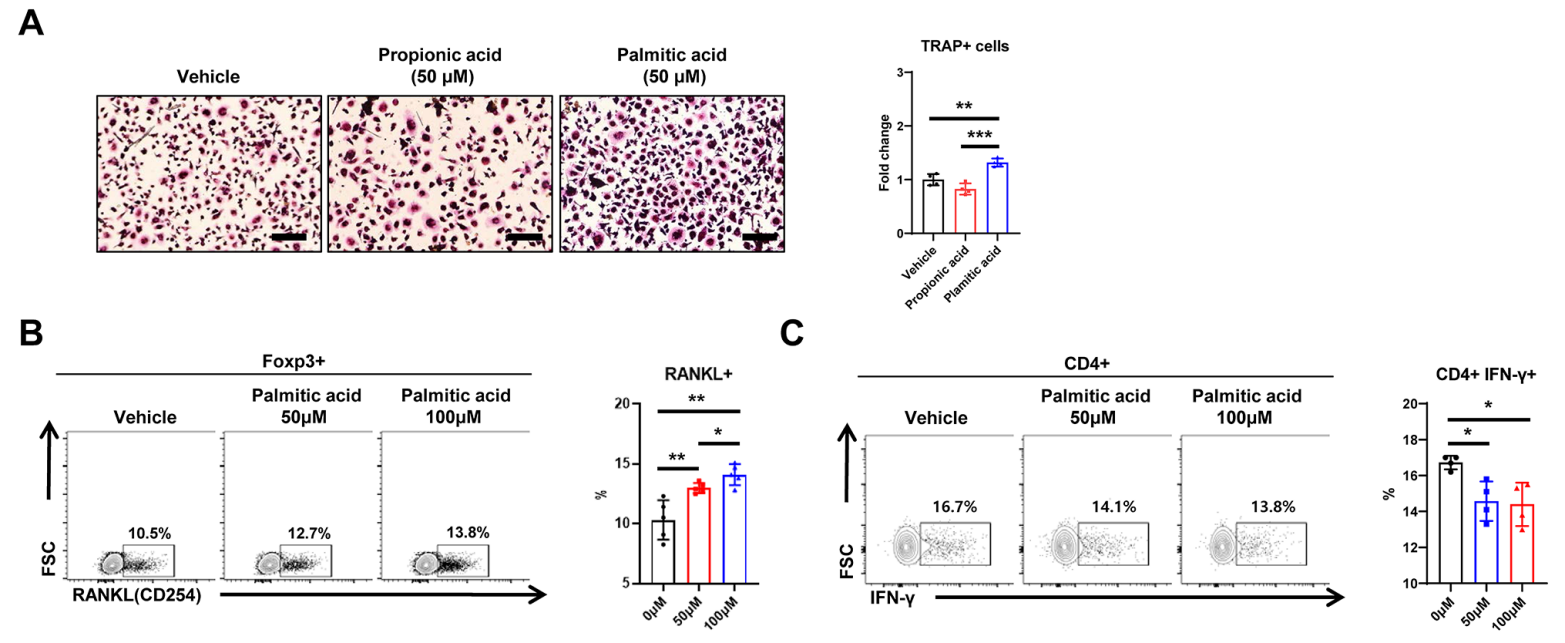
Treatment with palmitic acid increases the number of osteoclasts and promotes production of RANKL and IFN-γ in bone marrow T cells. **A** TRAP staining of osteoclasts and the number of TRAP-positive osteoclasts. Scale bar: 200 μm. **B** RANKL producing cells in Foxp3 + Treg populations treated with or without palmitic acid. **C** IFN-γ producing cells within the CD4 + T-cell populations treated with or without palmitic acid. Statistical significance was analyzed by one-way ANOVA. *, *P* < 0.05 and **, *P* < 0.01 compared with the indicated group

Reductions in lipolysis and fatty acid oxidation in BAT increase serum free fatty acids levels and decrease bone mass in BKO mice, suggesting that serum free fatty acids may directly or indirectly influence bone marrow cells. To determine the effects of fatty acids on bone marrow cells, we treated various types of bone marrow cells with different fatty acids. First, we treated bone marrow-derived osteoclasts with short-chain fatty acids (propionic acid) and long-chain fatty acids (palmitic acid). TRAP staining revealed no changes in the number of TRAP-positive osteoclasts after treatment with propionic acid compared with treatment with vehicle; however, a marked increase in the number of TRAP-positive osteoclasts was observed when the osteoclasts were treated with palmitic acid (Fig. 7A and B). Next, we examined for potential changes in bone marrow immune cell phenotypes following treatment with long-chain fatty acids by performing flow cytometry of bone marrow immune cells treated with palmitic acid for 24 h. Upon treatment with palmitic acid, the number of RANKL-expressing Tregs increased dose-dependently with the concentration of palmitic acid (50 µM and 100 µM), whereas the number of IFN-γ expressing CD4 + T cells significantly decreased compared with the vehicle-treated control cells (Fig. 7B and C). Taken together, these results suggest that, among the metabolites altered by the reduced lipolysis or fatty acid oxidation in BAT, long-chain fatty acids (palmitic acid) directly or indirectly regulate osteoclast differentiation, potentially leading to bone loss.

## Discussion

The relationship between ambient temperature and bone physiology is a complex and multifaceted one, with a growing body of evidence suggesting that environmental factors play a significant role in the regulation of bone metabolism. This study reveals that temperature is a critical determinant of bone remodeling, which has profound implications for our understanding of bone health and disease management.

The impact of temperature on bone physiology can also be seen through the lens of thermoregulatory metabolism. Mild cold stress, common in standard mouse housing conditions, is known to affect tumor growth rates, CD8 + T-cell and dendritic cell function, and the activity of immunosuppressive cells [31]. This underscores the importance of considering environmental factors when studying bone physiology and its interactions with the immune system. Our study also explored the temperature-dependent changes in bone marrow T-cell populations, shedding light on the immune-mediated mechanisms that may contribute to bone remodeling. We observed an increase in Tregs, which are known to suppress osteoclastogenesis, at thermoneutral temperatures (Fig. 2). This suggests that a warmer ambient temperature may protect against bone loss by modulating the immune environment within bone marrow. Conversely, at low temperatures, we noted an increase in the pro-resorptive T-cell populations (Fig. 2), which could contribute to the increased bone resorption observed at these temperatures. The results potentially highlight the importance of immune system interactions in mediating the effect of temperature on bone density.

An intriguing aspect of our study is the relationship between BAT activity and bone mass. BAT is known to play an important role in thermogenesis, the process via which heat is produced in organisms. Brown adipocytes are rich in mitochondria, which have a high oxidative capacity and contain UCP-1 in their inner membrane, [23] which is particularly active during cold exposure [22]. Numerous studies have reported a positive relationship between BAT activity and bone mass [4, 13–15, 17]. However, research into the mechanisms underlying the relationship between BAT and bone mass remains limited. Our data suggest that BAT activity correlates positively with bone mass, indicating that BAT protects against bone loss. This is further corroborated by the observation that mice lacking BAT have a reduced bone mass (Fig. 3). The thermogenic function of BAT, primarily driven by UCP-1 and mitochondrial oxidative phosphorylation, may be a key part of this protective mechanism. This is not only due to the thermogenerative properties of BAT but also to the metabolic activity associated with thermogenesis, which appears to have a systemic effect on bone tissue. Furthermore, our research suggests that BAT mitochondrial functions are crucial for bone health, particularly under cold stress conditions. Mice with compromised BAT mitochondrial functions exhibited accelerated bone loss when exposed to the cold, but not when exposed to thermoneutral conditions (Fig. 5). This underscores the importance of BAT mitochondrial integrity for the preservation of bone mass during environmental stress.

The metabolic mechanisms that mediate the effects of temperature variations on bone physiology are multifaceted. Our research suggests that lipolysis and β-oxidation in BAT play a pivotal role in modulating the levels of systemic metabolites. BAT is characterized by its abundance of mitochondria, [23] which possess a high capacity for oxidation and contain UCP-1 within their inner membranes. BAT activation is a complex physiological response to cold exposure, where norepinephrine-mediated signaling promotes lipolysis, releasing free fatty acids and glycerol. These substrates fuel mitochondrial beta-oxidation and, via UCP-1, facilitate thermogenesis instead of ATP production [32]. We found that reduced mitochondrial function in BKO mice was linked to decreases in OxPhos and fatty acid oxidation, leading to an increase in fatty acid-based metabolites. Additionally, there is evidence suggesting that reduced consumption of free fatty acids can lead to a decrease in lipolysis [33]. Moreover, the feedback mechanisms within adipocytes can regulate lipolysis through various pathways. An increase in intracellular free fatty acids can lead to their re-esterification into triglycerides, which effectively reduces the availability of free fatty acids for further lipolysis [34]. This indicates that mitochondrial dysfunction in BKO mice reduces the consumption of fatty acids through beta-oxidation in BAT, resulting in increased levels of both intra-BAT and circulating free fatty acids, and consequently, a reduction in lipolysis due to the abundance of fatty acids within BAT, thereby increasing circulating serum free fatty acids. (Fig. 6). Furthermore, among the increased metabolites, long-chain fatty acids not only increased the differentiation of osteoclasts as evidenced by increased TRAP staining (Fig. 7A and B), but also induced changes in cytokine expression in T cells, such as RANKL expression in Tregs and IFN-γ expression (Fig. 7), thereby potentially exerting indirect control over osteoclast regulation. However, our study is not without its limitations. Although we observed that housing temperature affected bone mass and BAT confers protection against bone loss induced by cold exposure, the mechanisms responsible for cold-induced bone loss were not clearly identified in this study. Additionally, RANKL is produced by various cell types, including T cells, B cells, epithelial cells, keratinocytes, endothelial cells, synovial fibroblasts, osteoblast precursors, mature osteoblasts, and osteocytes. [35] However, we hypothesize that circulating RANKL in the blood is attributable to RANKL-expressing Tregs, we were unable to quantitatively confirm this in the present study.

In conclusion, chronic cold exposure is associated with alterations in immune cells and is linked to bone loss. BAT plays a crucial role in protecting against bone loss induced by cold exposure, and this protective effect of BAT is linked to mitochondrial fatty acid oxidation and lipolysis-driven thermogenesis, as well as the consumption of long-chain fatty acids. Mitochondrial dysfunction in BAT is known to lead to an increase in systemic long-chain fatty acid levels, Our in vitro experiments support these findings, indicating that long-chain fatty acids can directly and indirectly upregulate osteoclasts. in turn promoting osteoclastogenic T-cell activity and bone loss.

### Electronic supplementary material

Below is the link to the electronic supplementary material.

Supplementary Material 1

Supplementary Material 2

Supplementary Material 3

Supplementary Material 4

Supplementary Material 5

Supplementary Material 6

## Data Availability

All data generated or analyzed during this study are included in this published article and its supplementary information files. All primary data will be shared by the lead contact upon request.
