# Supplementary material for: Brown fat-specific mitoribosomal function is crucial for preventing cold exposure-induced bone loss
Source: Cell Mol Life Sci. 2024 Jul 27;81(1):314. doi: 10.1007/s00018-024-05347-4 (PMC11335241; doi:10.1007/s00018-024-05347-4)
Supplement: Supplementary file 1 — Supplementary Material 1 [file 18_2024_5347_MOESM1_ESM.docx]

**Supplemental information**

**Brown fat-specific mitoribosomal function is crucial for preventing cold exposure-induced bone loss**

Jingwen Tian^1,2#^, Ji Sun Moon^1,2^, Ha Thi Nga^1,2^, Ho Yeop Lee^1,2^, Thi Linh Nguyen^1,2^, Hyo Ju Jang^1,2^, Daiki Setoyama^3^, Minho Shong^4^, Ju Hee Lee^2^, Hyon-Seung Yi^1,2*^

^1^Laboratory of Endocrinology and Immune System, Chungnam National University School of Medicine, Daejeon 35015, Republic of Korea. ^2^Department of Internal Medicine, Chungnam National University School of Medicine, Daejeon 35015, Republic of Korea. ^3^Department of Clinical Chemistry and Laboratory Medicine, Kyushu University Hospital, Fukuoka, Japan. ^4^Graduate School of Medical Science and Engineering, Korea Advanced Institute of Science and Technology, Daejeon, Republic of Korea.

*Corresponding author. Research Center for Endocrine and Metabolic Diseases, Chungnam National University Hospital, Chungnam National University School of Medicine, Daejeon 35015, Republic of Korea, **Tel:** +82-42-280-6994; **Fax:** +82-42-280-6880; **E-mail:** jmpbooks@cnu.ac.kr

**
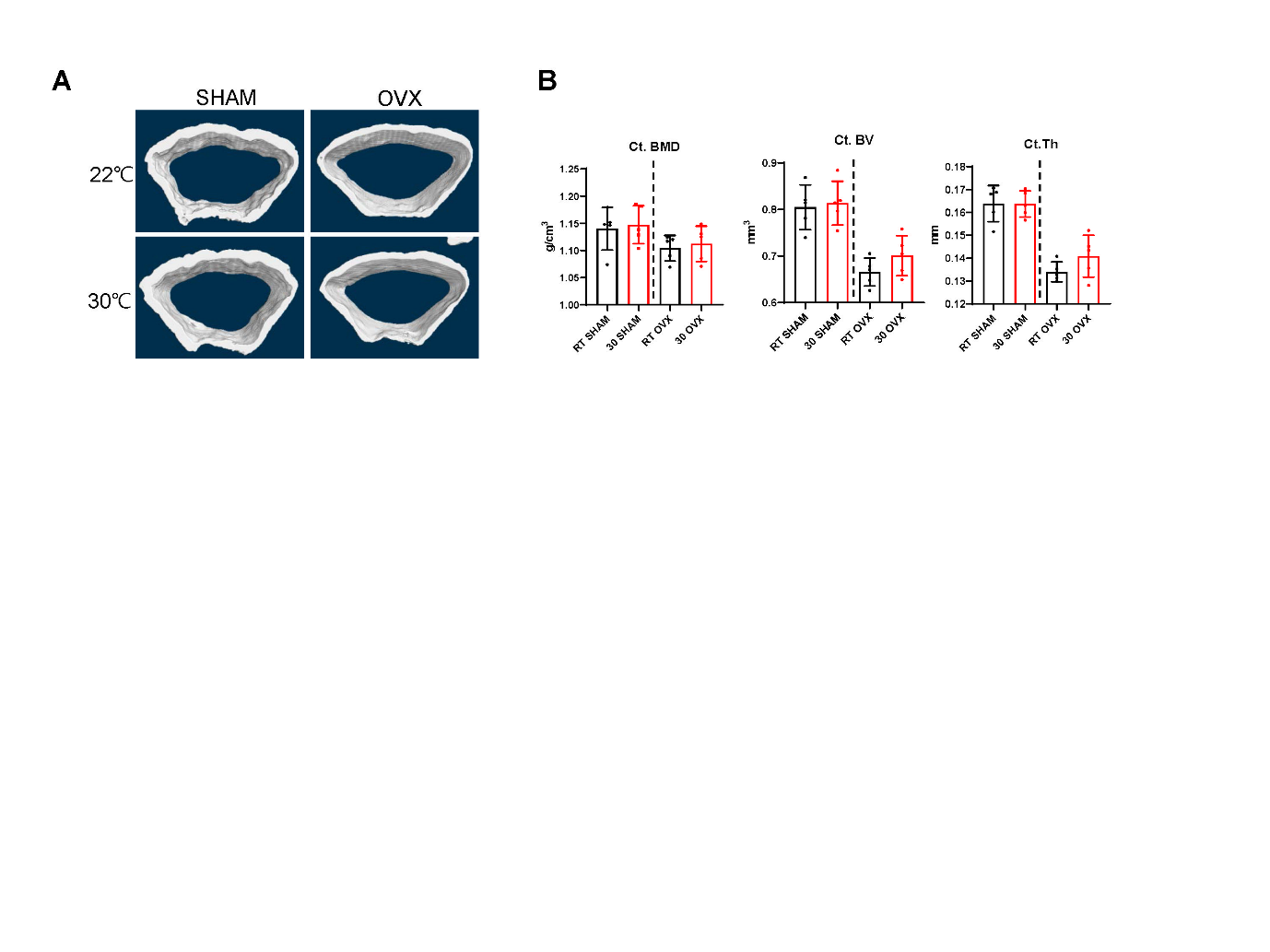
Supplementary figures and Supplementary figure Legends**

**Supplementary Figure 1** **A** Illustrative micro-CT images showing the cortical areas in the lower section of the femur. **B** Measurements of Ct.BMD, Ct.BV, and Ct.Th in the femur.


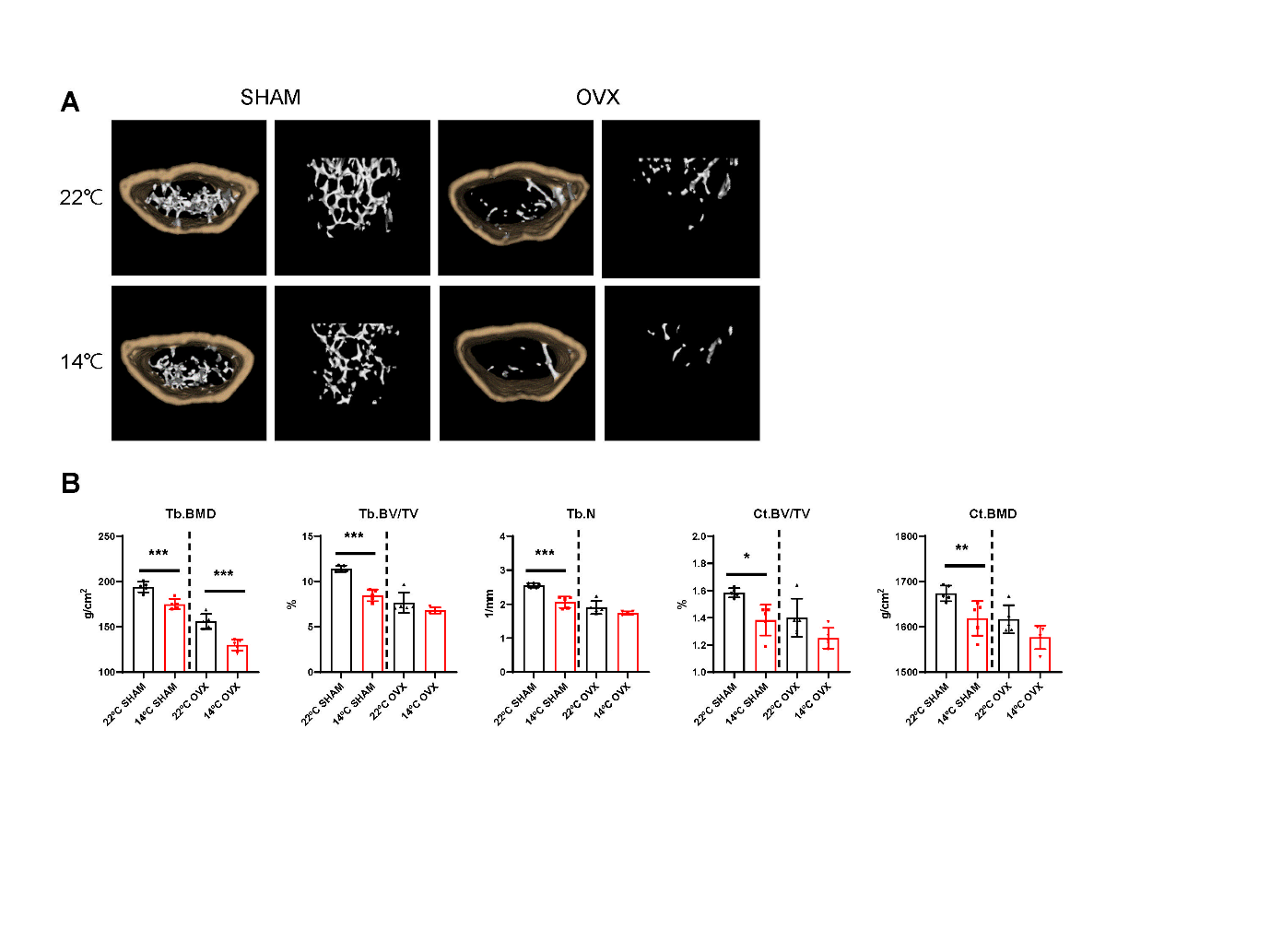


**Supplementary Figure 2** **A** Representative micro-CT images of cortical and trabecular regions in the distal femur. **B** Measurement of bone parameters of the distal femur analyzed by micro-CT. To determine the statistical relevance of the findings, unpaired t-tests were used for analysis. *, P < 0.05, **, P < 0.01, and ***, P < 0.001.


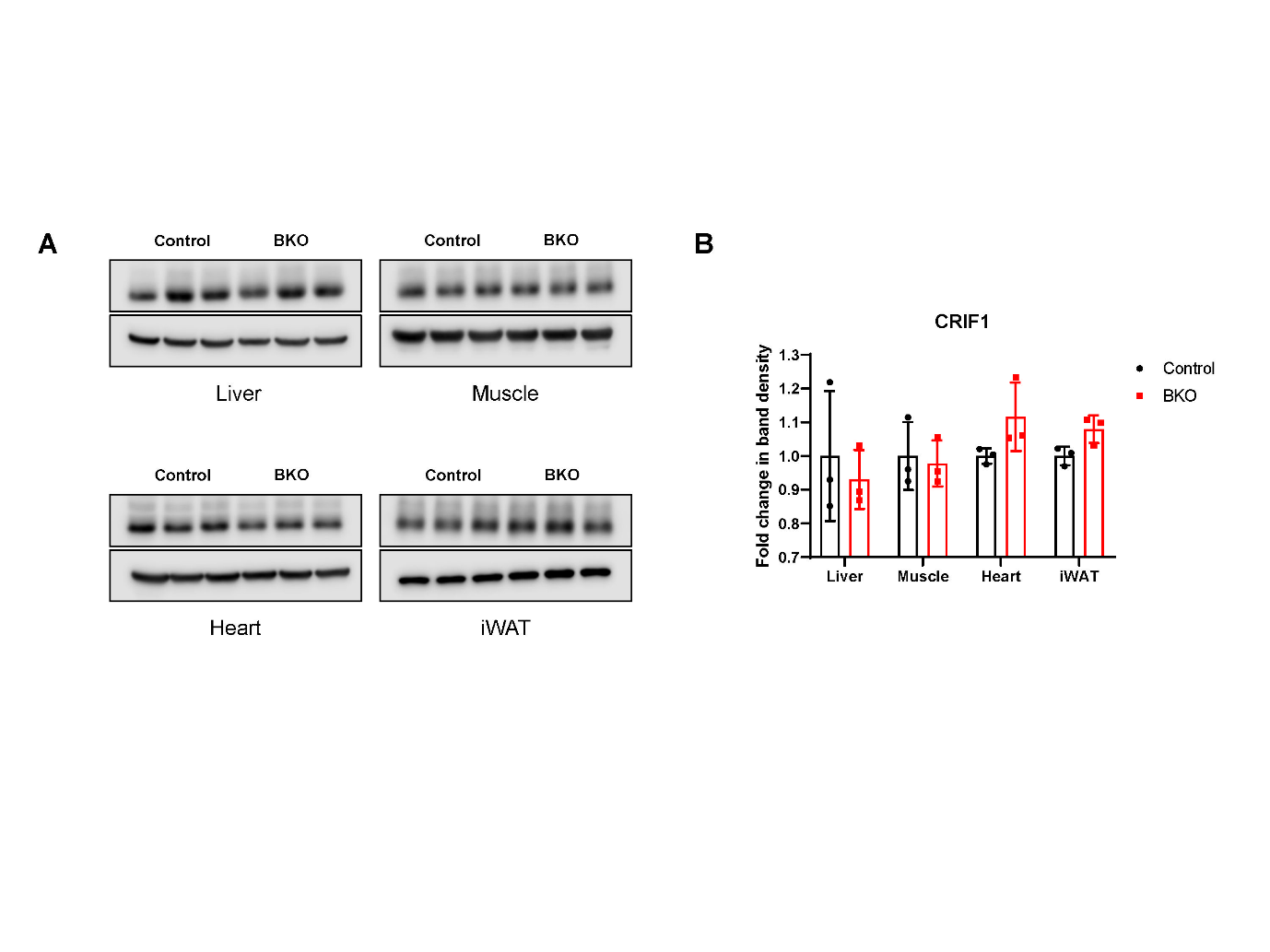


**Supplementary Figure 3** **A,B** Representative western blots and band density measurements of CRIF1 in liver, muscle, heart and iWAT isolated from control and BKO mice housed at 22°C at 20 weeks old. To determine the statistical relevance of the findings, unpaired t-tests were used for analysis. *, P < 0.05 and **, P < 0.01.


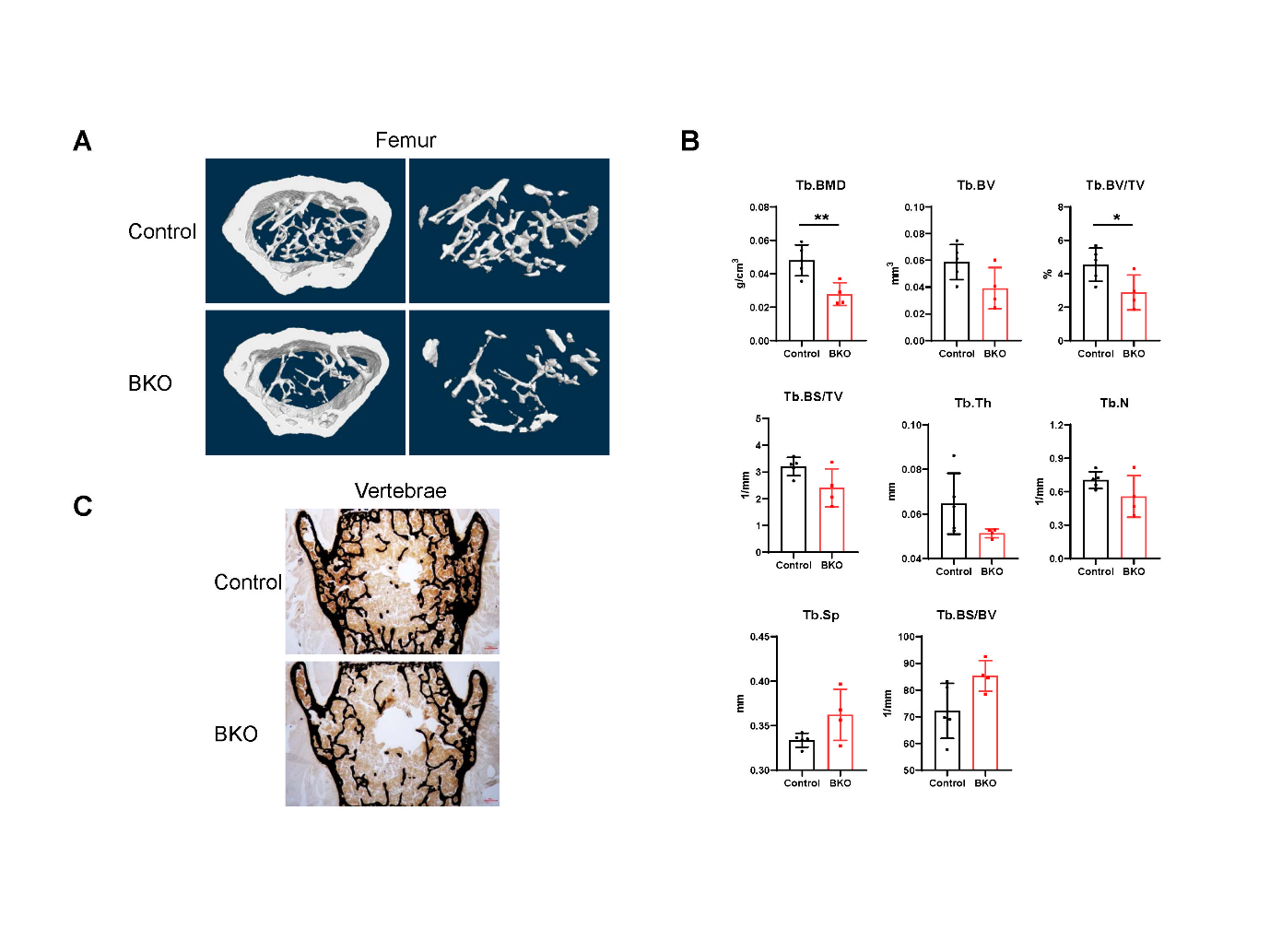


**Supplementary Figure 4** **A** Representative micro-CT images of cortical and trabecular regions in the distal femur of control and BAT-specific mitochondrial dysfunction (BKO) mice at 20 weeks of age. **B** Measurements of Tb.BMD, Tb.BV, Tb.BV/TV, Tb.BS/TV, Tb.Th, Tb.N, Tb.Sp, and Tb/BS/BV. **C** Von Kossa staining of undecalcified sections of the vertebrae of BKO and wild-type control mice. To determine the statistical relevance of the findings, unpaired t-tests were used for analysis. *, P < 0.05 and **, P < 0.01.


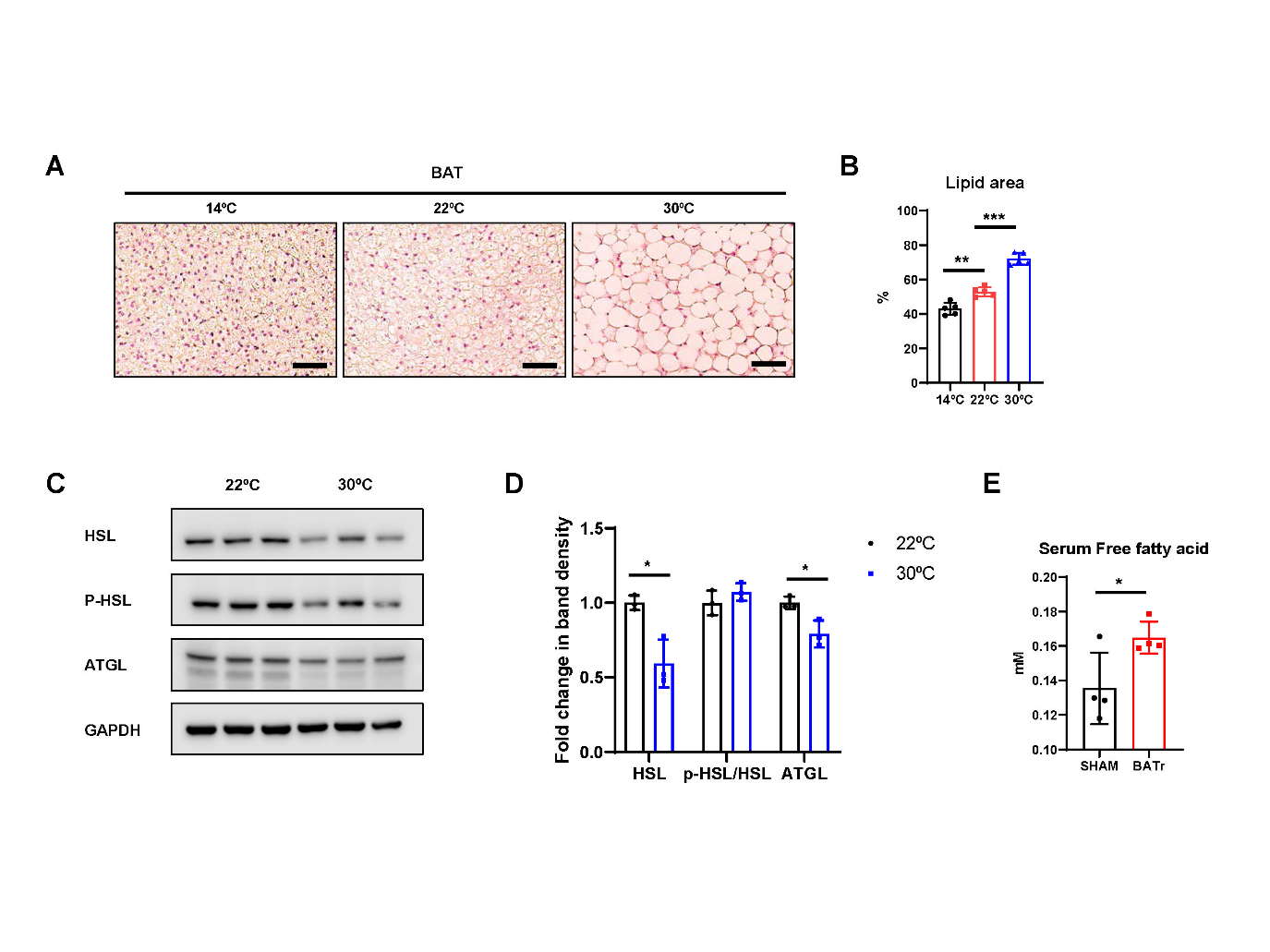


**Supplementary Figure 5** **A** Representative H&E staining images of BAT across different housing temperatures. **B** Statistical analysis of lipid area. **C, D** Representative western blots and band density measurements of HSL, p-HSL, and ATGL in BAT isolated from mice housed for 12 weeks at 22°C or 30°C. **E** Quantification of serum free fatty acids in mice housed at 22°C for 12 weeks following post-surgical removal of BAT. To determine the statistical relevance of the findings, unpaired t-tests were used for analysis. *, P < 0.05 and **, P < 0.01.

**Supplementary Table 1. List of primers and their sequences**

| Primer | Forward sequence | Reverse sequence |
| --- | --- | --- |
| *18s* | CTG GTT GAT CCT GCC AGT AG | CGA CCA AAG GAA CCA TAA CT |
| *Cpt1a* | TAT AAC AGG TGG TTT GAC | CAG AGG TGC CCA ATG ATG |
| *Cpt1b* | TCG CAG GAG AAA ACA CCA TGT | AAC AGT GCT TGG CGG ATG TG |
| *Acadm* | TGA CGG AGC AGC CAA TGA | TCG TCA CCC TTC TTC TCT GCT T |
| *Ppara* | AGA AGT TGC AGG AGG GGA TT | TTG AAG GAG CTT TGG GAA GA |
| *Pgc1a* | TCT CAG TAA GGG GCT GGT TG | AGC AGC ACA CTC TAT GTC ACT C |

**Supplementary Table 2. Antibody list (Western blot, immunohistochemistry, and FACS)**

| Name | Company | Cat. No |
| --- | --- | --- |
| UCP-1 | Abcam | Ab10983 |
| Total OXPHOS cocktail | Abcam | Ab110412 |
| CRIF1 | Santa Cruz | Sc-374122 |
| HSL | Cell Signaling | 4107S |
| p-HSL | Cell Signaling | 45804 |
| ATGL | Cell Signaling | 2138S |
| GAPDH | Cell Signaling | 2118S |
| β-ACTIN | Cell Signaling | 4967S |
| SDHB | Cell Signaling | 92649S |
| PE-eFl610 anti-CD3 | Invitrogen | 61-0031-82 |
| PE anti-NK1.1 | BD Biosciences | 557391 |
| PerCP-eF710 anti-CD4 | Invitrogen | 46-0041-82 |
| AlexaFlour700 anti-CD8a | BioLegend | 100730 |
| SB436 anti-CD25 | Invitrogen | 62-0251-82 |
| APC anti-FOXP3 | Invitrogen | 17-5773-82 |
| PE anti-CD254 | BioLegend | 510006 |
| APC anti-IFN gamma | Invitrogen | 17-7311-82 |
| PE anti-IL-17A | Invitrogen | 12-7177-81 |
